# Thyroid-Stimulating Hormone-Secreting Pituitary Adenoma: Two Cases With Challenging Diagnosis and Management

**DOI:** 10.1155/crie/5103475

**Published:** 2025-04-14

**Authors:** Elodie Gruneisen, Juan Andres Rivera

**Affiliations:** ^1^Division of Endocrinology, McGill University Health Centre, Montréal, Canada; ^2^Division of Endocrinology, Diabetology and Metabolism, Department of Medicine, Centre Hospitalier Universitaire Vaudois (CHUV), Lausanne, Switzerland

## Abstract

**Background:** Thyroid-stimulating hormone (TSH)-secreting pituitary adenomas (TSHomas) are very rare pituitary tumors causing central hyperthyroidism. Most are macroadenomas (≥ 10 mm) with local and systemic comorbidities at diagnosis. The atypical changes in thyroid function tests (TFTs) may be subtle and are often initially missed, while over-secretion of other pituitary hormones is often present. Somatostatin analogs (SSAs) are the recommended first-line medical therapy for these lesions. We report two cases of TSHomas successfully managed with a dopamine agonist (DA) therapy, alone or following transsphenoidal surgery (TSS).

**Case Presentation:** A 47-year-old man presented with significant weight loss, fatigue, and muscle weakness. He was found to have hyperprolactinemia, secondary adrenal insufficiency (AI), and central hypogonadism, which led to the discovery of a 3 cm invasive pituitary adenoma. Additional tests showed an increased IGF1, TSH, and free T4. A Pit-1 multihormonal tumor was documented on pathology after partial resection by TSS. Persistent hyperprolactinemia and central hyperthyroidism responded to DA therapy, as the patient refused therapy. A 66-year-old man with a history of anxiety, hypertension, coronary artery disease, atrial fibrillation, and thyroid nodules, was consulted for severe dizziness and was found to have a 2.4 cm pituitary adenoma on a head CT scan. Lab records showed a progressive supranormal free T4 and TSH increase over the preceding five years. He refused surgery and had an excellent clinical and biochemical response to DA treatment.

**Conclusion:** Prompt detection of central hyperthyroidism by monitoring and correctly interpreting TFT over time is essential for early diagnosis and optimal management of TSHomas. TSH-secreting adenomas may respond to DA therapy.

## 1. Introduction

Thyroid-stimulating hormone (TSH)-secreting pituitary adenomas (thyrotropinomas or TSHomas) are rare pituitary tumors that cause secondary or central hyperthyroidism. They represent 0.5%–2% of all functional pituitary adenomas, and their prevalence in the general population is estimated at one case per million[1], without gender difference. Although they may remain underdiagnosed, there have been an increasing number of reported cases over time due to improvements in imaging and laboratory assays. About 30% of TSHomas are multisecretory Pit-1 adenomas (TSH with PRL and/or GH) [2, 3]. Historically, the majority of reported cases (80% of cases) are macroadenomas (≥10 mm) [4, 5], with symptoms of expanding tumor mass (visual field defects, headache, hypopituitarism). Early detection, therefore, is essential to optimize their management. Transsphenoidal surgery (TSS) is the mainstay of therapy in the majority of cases. When medical therapy is necessary, somatostatin analogs (SSAs) are the recommended first choice. A few reported cases of TSHomas have responded to dopamine agonist (DA) therapy. Here, we report two cases with different clinical presentations, challenging diagnoses, and therapeutic approaches that resulted in the early use of DAs with good results.

## 2. Cases Presentation

### 2.1. Case 1

**Figure 1:**
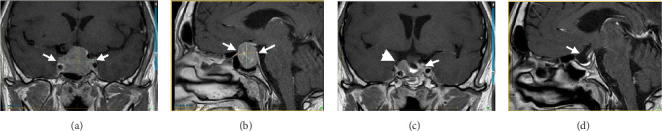
Case 1: (A, C) Coronal and (B, D) sagittal postgadolinium T1-MR images of the pituitary macroadenoma with extension to the cavernous sinus (arrows), in 2017 (A, B) before transsphenoidal surgery and in 2019 (C, D) two years after surgery. Note the significant reduction in tumor mass with residual tumor mainly in the right cavernous sinus (larger arrowhead in C).

A 47-year-old man of Portuguese extraction presented in 2015 with headaches, anorexia, about 10% body-weight loss, fatigue, muscle weakness, and decreased libido. His medical history was limited to “urinary bladder dysfunction” and prostate stones, for which he was taking tamsulosin. He denied using other medications or nonprescribed drugs or supplements. He was a 20-pack-year smoker. His family history was unremarkable. His initial laboratory evaluation showed hyperprolactinemia of 59 µg/L (ref. 2.6–13.1). An MRI of the sella revealed a 3.2 × 2.7 × 2.1 cm pituitary adenoma with cavernous sinus invasion (Figure 1). On exam, his BP was 96/65 mmHg, HR 72 bpm, and BMI 23 kg/m^2^. His facial features were coarse, and he had large, thick hands and feet. He reported all to be in keeping with familial traits. He had no goiter or thyroid nodules; his visual fields were normal. The initial GH was 2.87 ng/mL (ref. 0.01–1.70), and IGF1 was 31 nmol/L (ref. 14.7–42.4). Bioavailable testosterone was low at 0.15 nmol/L (ref. 3.6–11), with LH 0.8 IU/L, and FSH 1 IU/L. TSH was mildly elevated at 5.35 mIU/L (ref. 0.40–4.40) and free T4 at 23.1 pmol/L (ref. 8–18). On a 1-µg cosyntropin stimulation test, the peak cortisol was 358 nmol/L (ref. > 420 nmol/L). ACTH was 4.78 pmol/L (1.6–13.9). These results were consistent with secondary adrenal insufficiency (AI), hypogonadotropic hypogonadism, and secondary hyperthyroidism, possibly due to a TSHoma. TSH or free T4 assay interference was ruled out with alternative assay platforms and remeasuring in pre-treated samples. The alpha-subunit (α-SU) of glycoprotein hormones was borderline at 0.5 ng/mL (ref. < 0.5). Sex hormone-binding globulin (SHBG) was elevated at 111 nmol/L (reference range 13–89), suggesting hyperthyroidism in addition to liver disease. He was started on cortisol and testosterone replacement, and neurosurgery was consulted. Repeated labs (six months after the initial workup) showed now an elevated IFG-1 at 86 nmol/L (ref 14.7–42.4) and a basal GH at 5.52 µg/L (ref. 0.01–1.70). During an oral glucose challenge, the GH trough was high at 2.74 µg/L (ref. < 0.4), in line with neoplastic GH elevation. He gained 12 kg (26.5 Lb; likely from high GH) and reported thicker hands, fingers, and coarser facial features. He was hyperthermic, and a fine distal tremor was now evident. A multihormonal-secreting tumor (GH/TSH/prolactin) was suspected. He was started on quinagolide, and later, the long-acting subcutaneous SSA, lanreotide was added. In November 2017, he underwent TSS and had a partial resection of the tumor. Early TSH, FT4, GH, and cortisol normalization was documented (Table 1). Pathology reported a Pit-1 adenoma, which stained positive for GH (sparsely granulated pattern) and, in rare cells, for TSH. Immunohistochemical staining was negative for prolactin, FSH, and LH.

The patient remained well until the end of 2020, when biochemical recurrence was documented (Table 1), with a prolactin increase to 59 µg/L (ref 2.6–13.1) and a high free T3 at 6.58 pmol/L (ref. 3.8–6). IGF-1 remained normal at 27.3 nmol/L (ref. 14.7–42.4). This time, the patient refused therapy with SSA based on previous side effects, so a cabergoline trial was started, resulting in prolactin suppression (0.3 µg/L) and a modest reduction in free T3 on doses up to 2 mg per week. The patient remains asymptomatic and refuses injectables. Therapeutic options, including repeat surgery and radiotherapy, remain under consideration.

### 2.2. Case 2

**Figure 2:**
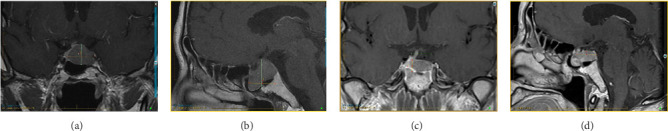
Case 2: (A, C) Coronal and (B, D) sagittal T1-FSE MRI selected images, (A, B) pretreatment in 2019 and (C, D) after cabergoline therapy in 2022. Tumor dimensions pretreatment were 2.18 cm × 2.38 cm × 2.17 cm vs. 1.71 cm × 0.96 cm × 2.02 cm during treatment.

A 66-year-old Chinese man was consulted in 2019 because of severe dizziness and left hemibody dysesthesia. A CT scan of the head revealed a sellar mass. An MRI of the sella confirmed a 2.18 × 2.38 × 2.17 cm adenoma extending to the left cavernous and sphenoid sinuses (Knosp 2, Figure 2). He had a history of hypertension, coronary artery disease, atrial fibrillation, and thyroid nodules. He had undergone a Whipple's procedure a year prior at another institution for a benign pancreatic cyst. His medications include sotalol, flecainide, rivaroxaban, celecoxib, pantoprazole, pancreatic enzymes, and vitamins B and C. He reported persistent anxiety for 4–5 years, an ~12% body weight loss over two years, and chronic palpitations. On exam, visual fields were normal. He had a small goiter (~50 g). BP was 138/76 mmHg and HR 53 bpm. Laboratory tests showed mildly elevated prolactin at 29.9 µg/L (ref 2.6–13.1), TSH 5.7 mIU/L (ref 0.4–4.40), free T4 25.8 pmol/L (ref 8–18), and free T3 6.44 pmol/L (ref. 3.8–6). Morning cortisol was 450 nmol/L. IGF1, LH, FSH, and testosterone were normal. He had been followed by a thyroid surgeon for over a decade because of a 2 cm right lobe solid thyroid nodule, which had been eventually proven benign. In retrospect, laboratory records showed a progressive free T4 and TSH increase over the previous five years, consistent with worsening central hyperthyroidism (Table 2). TSH or FT4 assay interference was ruled out. A TSH-secreting pituitary macroadenoma with cavernous sinus invasion and stalk compression was diagnosed. The patient refused TSS and intramuscular injections (octreotide and lanreotide). He was started on cabergoline in 2020, and the dose was progressively increased from 0.25 mg biweekly to the current 1.25 mg biweekly, which resulted in clinical and biochemical euthyroidism and tumor shrinkage on MRI (see Figure 2 and Table 2).

## 3. Discussion

The cases discussed here are illustrative of the spectrum of clinical presentation of TSH-secreting pituitary adenomas. The clinical symptoms of hyperthyroidism are usually present in TSHomas but may be mild and masked by concomitant hormonal abnormalities. In Case 1, AI and hypogonadism symptoms were present, partly obscuring hyperthyroidism symptoms. The initial clinical presentation changed over time with GH over secretion, which was later confirmed during follow-up. In Case 2, the diagnosis was delayed despite abnormal thyroid function test results and symptoms of hyperthyroidism during the follow-up for a thyroid nodule by a surgeon. In all cases of TSHoma, the diagnostic hallmark is a persistently high normal, or raised free T4 and T3 with inappropriately normal or elevated TSH. This finding should be followed by a sequence of steps to rule-out conditions associated with similar TFT results. The first step is to exclude assay interferences, either from commonly used medications (furosemide, heparin, beta-blockers, or amiodarone) or from heterophilic antibodies, which can nonspecifically bind assay anti-T3, anti-T4, and/or anti-TSH antibodies, leading to falsely high results [5]. No interfering medications were present in any of our cases. The presence of heterophilic antibodies can be ruled out by one or more of the following methods: using blocking agents such as PEG or non-immune animal sera; using an alternative assay platform or with serial sample dilutions. We used alternative assay platforms in our cases.

The second step is to rule out other rare conditions with a similar TFT profile, that is, thyroid hormone resistance syndrome (THRS) or familial dysalbuminemic hyperthyroxinemia (FDH). The estimated prevalence of THRS is 1/40,000−19,000 live births [6]. In THRS, a loss-of-function mutation in the thyroid hormone receptor beta (THRB) gene results in impaired tissue sensitivity to thyroid hormone. A new set point is present since birth whereby FT4 and FT3 levels are permanently high without TSH suppression. FDH, a major cause of inherited euthyroid hyperthyroxinemia in Caucasians, has a prevalence of ~1/10,000 subjects, particularly those of Hispanic origin (1.0%−1.8%) [7]. In FDH and other alterations in thyroid hormone binding proteins, a mutation results in greater affinity for thyroid hormone, resulting in a much higher total T4 and T3 concentration. When the free thyroid hormones are measured using immunoassays, interference may occur, and high FT4 and FT3 levels are measured. In THRS, there may be some hyperthyroid symptoms, primarily cardiovascular and neurological, as in both these tissues, the THR alpha (which is not mutated) is more prevalent. A family history of a similar TFT profile in first-degree relatives and lifelong symptoms of mixed hypo and hyperthyroid symptoms would heighten suspicion of THRS. In FDH, the patient is euthyroid. These conditions can be excluded based on historical records showing normal TFT shifting over time. This was the case for our Case 2.

Increased circulating levels of α-subunit (in 70% of TSHomas) and α-subunit/TSH molar ratio (>1.0, in 80% of TSHomas) from unbalanced α-subunit formation, in the context of a pituitary adenoma, also support the diagnosis of TSHoma [8]. Additionally, markers of end-organ effects of TH (SHBG, ferritin, alkaline phosphatase) are usually elevated in patients with TSHoma but normal in patients with THRS or FDH [9]. However, these latter have demonstrated a lack of specificity: for example, SHBG levels could be increased by exogenous estrogen treatment, liver failure, insulin resistance, obesity, and increased growth hormone [10]. In Case 1, the elevation of the alpha subunit and the therapeutic response to DAs excluded THRS.

Finally, dynamic tests (T3-suppression and TRH-stimulation), although not required to establish the diagnosis of TSHoma, can be done in selected cases to support the diagnosis. Although there is a lack of uniform reference data for normal controls and patients with THRS, and its use is limited to young patients without cardiac disease, there is typically no suppression of TSH in response to oral T3 administration (the T3 suppression test) in TSHomas. Additionally, in contrast with patients with THRS, there is typically no increase in TSH or α-subunit after intravenous TRH (the TRH stimulation test) in patients with TSHomas [11]. Unfortunately, TRH is not available in all centers, including ours.

Medical therapy aiming to control hyperthyroidism is usually initiated as soon as the diagnosis is confirmed. TSS by an expert pituitary neurosurgeon should be subsequently considered as it is the preferred definite treatment for TSHomas whenever feasible. The rate of cure with TSS varies in different reports, but in general, euthyroidism is obtained in as many as 75%–85% of cases. However, resection could be incomplete in some patients due to high tumor volume and local invasion.

First-generation SSA, such as octreotide and lanreotide, which inhibit TSH production from TSHomas through the somatostatin receptors (SSTRs) 2 and 5 are the most frequently recommended medical therapy, as they lead to a TSH reduction in about 80%–90% of cases [9]. In somatotropinomas, resistance to SSA has been related to high expression of the truncated isoform of the SSTR5 (sst5TMD4) [12]. It has also been shown that TSHomas that respond poorly to SSA have a lower SST5/SST2 and SST/DR2 expression ratio and respond better to DA [13].

Dopamine receptor subtype 2 (DR2) is expressed in all pituitary normal and tumoral cells [14]. DR2 is particularly highly expressed in lactotroph cells, accounting for the effectiveness of DA in prolactin-co-secreting adenomas [13, 15], as in our Case 1. In published case reports and series, DA, including bromocriptine and cabergoline, have been shown to be useful in achieving euthyroidism and tumor shrinkage in TSHomas [16–18] as illustrated in our cases.

In Case 2, the diagnosis was consistent with a pure TSHoma, with mildly elevated prolactin level in the context of compressed pituitary stalk and a suppressed dopamine pathway. This patient declined surgery or injectable therapy but accepted to take oral treatment with a DA. Our experience supports that DA therapy is a viable option to treat TSHomas when surgery is contraindicated, not curative, or declined.

## 4. Conclusion

These cases illustrate how the definitive diagnosis of TSHomas in endocrine clinical practice can be convolute, given its rarity, in contrast with the frequency of transient atypical TFT and the masking effect of other more frequent and clinically obvious associated hormonal abnormalities. The differentiation between TSHomas, THRS, incidental FDH, or assay interference may also be challenging and is based on a combination of clinical, biochemical, and radiographic criteria.

Awareness of clinical context, judicious follow-up, and skillful interpretation of TFTs over time will help reach a prompt, correct diagnosis, and select the optimal treatment approach to prevent tumor-growth complications. DA treatment is a convenient therapeutic option in selected cases.

## Figures and Tables

**Table 1:** Case 1: Bloodwork before and after medical and surgical treatment.

Intervention		Qui-SSA (Feb 2017)		TSS (Nov 2017)				Cab (Oct 2021)	
Date	Aug 2016	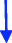	Aug 2017	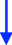	Mar 2018	Dec 2020	Jul 2021	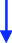	Jan 2023
TSH mIU/L (0.40–4.40)	**5.35**		1.42		2.3	1.7	**2.18**		**2.57**
Free T4 pmol/L (8–18)	**23.1**		15.3		10.2	14	**17**		**14.2**
Free T3 pmol/L^a^ (3.8–6)	—		—		—	—	**6.58**		**6.05**
Total T3 nmol/L^a^ (1.34–2.73)	**3.6**		2.68		—	—	—		—
Prolactin mcg/L (2.6–13.1)	**44.6**		0.9		20.7	**43.9**	**59**		**0.3**
IGF1 nmol/L (14.7–42.4)	31.3		**81.6**		35.5	24.4	27.3		20.7
GH mcg/L (0.01–1.7)	**2.87**		—		4.71	0.42	**1.81**		0.62

*Note*: T3, triiodothyronine; T4, thyroxine. Abnormal values are defined in bold.

Abbreviations: Cab, carbergoline; IGF1, insulin-like growth factor 1; Qui, quinagolide; SSA, somatostatin analog; TSH, thyroid-stimulating hormone; TSS, trans-sphenoidal surgery.

^a^Use total T3 as free T3 became unavailable at our center during this period.

**Table 2:** Case 2: Bloodwork before and after dopamine agonists (DAs) treatment.

Intervention			1^st^ MRI	1^st^ visit to Endocrinology	Cab (May 2020)		
Date	July 2016	May 2018	December 2019	February 2020	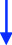	October 2020	September 2023
TSH mIU/L (0.40–4.40)	3.47	3.64	**4.54**	**5.7**		2.49	3.55
Free T4 pmol/L (8–18)	13.2	14.6	**18.1**	**25.8**		9.4	11
Free T3 pmol/L (3.8–6)	—	—	—	**6.44**		4.46	3.23
Prolactin mcg/L (2.6–13.1)	—	—	**13.6**	**29.9**		**< 0.3**	**< 0.3**

*Note*: T3, triiodothyronine; T4, thyroxine. Abnormal values are defined in bold.

Abbreviations: Cab, cabergoline; TSH, thyroid-stimulating hormone.

## Data Availability

The data used to support the findings of this study are available from the corresponding author upon request.
